# Assessment of patients’ satisfaction and associated factors among outpatients received mental health services at public hospitals of Mekelle Town, northern Ethiopia

**DOI:** 10.1186/s13033-018-0217-z

**Published:** 2018-07-11

**Authors:** Haftom Desta, Tesfay Berhe, Solomon Hintsa

**Affiliations:** 1 Department of Psychiatry, College of Health Science, Aksum University, P. O. Box: 298, Aksum, Ethiopia; 2 School of Public Health, College of Health Science, Aksum University, P. O. Box: 298, Aksum, Ethiopia; 3 Department of Human Nutrition, School of Public Health, Aksum University, Aksum, Ethiopia; 4 Department of Epidemiology and Biostatistics, School of Public Health, Aksum University, Aksum, Ethiopia

**Keywords:** Satisfaction, Factors, Psychiatry, Outpatient, Mental health services

## Abstract

**Background:**

Satisfaction is the psychological state that results from confirmation or disconfirmation of expectations with reality. Patients’ satisfaction is a healthcare recipient’s reaction to salient aspect of the contexts, process and result of their service experience. The aim of this study was to assess patient satisfaction and associated factors among outpatients receiving mental health services at public hospitals in Mekelle town.

**Objectives:**

To assess patient satisfaction and associated factors among outpatients receiving mental health services at public hospitals in Mekelle town, northern Ethiopia.

**Methods:**

An institution based cross-sectional study was conducted among 415 outpatients receiving mental health services at public hospitals in Mekelle town from September 2013 to August 2014. The data were collected using standardized, structured pre-tested questionnaire. Participants were selected by systematic random sampling technique. Satisfaction rate was examined with the client satisfaction questionnaire (CSQ-8), having four responses ranging from poor to very good. Descriptive summary using percentages, frequency and graph were used to present study results. Multivariate logistic regressions with 95% confidence interval (CI) were used to assess the strength and *p-*value < 0.05 was used to indicate the significance of the association.

**Results:**

A total of 415 respondents were enrolled, with a response rate of 100% and magnitude of satisfaction of 72%. The predictors associated with patient satisfaction were higher education (AOR = 0.34; 95% CI 0.24, 0.97), longer waiting time (AOR = 0.01; 95% CI 0.002, 0.07), having a diagnosis of psychosis (AOR = 2.36; 95% CI 1.41, 5.72) were significantly associated with satisfaction.

**Conclusion and recommendation:**

More than one-four of patients receiving mental health services were dissatisfied with the service they received. Improvement in accessibility and availability of drugs, minimizing consultation time (< 45 min) or increasing number of OPD units are important to improve satisfaction.

## Background

Satisfaction can be defined as the extent of an individual’s experience compared with his or her expectations. It is the psychological state that results from confirmation or disconfirmation of expectations with reality [1]. Patients’ satisfaction is a healthcare recipient’s reaction to salient aspects of the contexts, process, and result of their service experience [2]. Client satisfaction with treatment process may be both influences and be influenced by treatment outcomes. Clients who are not satisfied with a service may have worse outcomes than others. Because, they miss more appointments, leave against advice or fail to follow on treatment plans [2, 3]. Patient satisfaction should be as indispensable to the assessment of quality as to the design and measurement of health care system [4].

Patients’ satisfaction, in particular, is subdivided into an orientation towards care and conditions of care. Orientation towards care represents the patient’s response from the use of a hospital in view of their hope and expectation which is measured by CSQ-8 (client satisfaction questionnaire). The conditions of care represents specific conditions including the method of treatment, the location of the care institution, the waiting time, the payment, the treatment results that are measured by CIC (community interest company questionnaire) [5].

Unlike clinical process measures, which are strictly facility centered, patient satisfaction is ‘patient-centered’ process measures. This is an excellent opportunity to involve patients in the process of evaluating the given service and program. Since they are the best source of information, their experiences often reveal how well a hospital system is operating and can stimulate important insight into kind of changes that are needed to close the chasm between the care provided and the care that should be provided. Patients should be allowed to define their own priority and evaluating their care accordingly, rather than having those criteria selected by other professionals [6].

Patient satisfaction is an integral component of health services. Studies of quality of medical care importance are increasing in importance as a component of health research. Consumer opinion of services is being taken into account in the assessment of quality. Thus evaluating a quality of medical care involves the measurement of its benefits to patients and community at large [7]. Patients’ ratings of their experiences and satisfaction with health services are a frequently used indicator of service quality [8]. However, there is a limited understanding of how psychiatric units contribute to patients’ perceptions of quality [9].

A study conducted in Geneva on patient satisfaction with psychiatric outpatient care reveal that the global satisfaction rate was 93.1%. Whereas the satisfaction level in Africa ranges 55–83%. In Ethiopia, the satisfaction level ranges 57.1–99% [10–18]. The most risk factors associated with patient satisfaction are age, educational status, waiting time, consultation duration, being an in-patient, having a diagnosis of psychosis, contact with services for more than 6 years, marital status, sex of relatives, perceived empathy, perceived technical competency, non-verbal communication and patient [16, 19–27].

There are limited studies about patient satisfaction and associated factors in the study area, so, the aim of this study is to determine the patient satisfaction level and associated factors among outpatients receiving mental health service in public health services of Mekelle town, northern Ethiopia.

## Methods

### Study design and area

Institution based cross-sectional study was conducted from September to August 2014. Tigray regional state has 1 referral hospital, 16 general hospitals, 218 health centers and 668 health posts. Mekelle Town is a capital city of Tigray region, located in 783 km north of Addis Ababa, Ethiopia. Mekelle Town has two public hospitals; Ayder referral hospital and Mekelle hospital.

### Study population

The sample size was determined using the formula for a single population proportion based on the assumptions 95% confidence level, 5% degree of precision and 57% the proportion of patient satisfaction [19] Taking this assumption ‘n’ became 374 and 10% non-response rate; the final sample size was 415. Systematic random sampling was used to reach the study unit using the sampling frame of patients attending each hospital psychiatric out patient department from patient registration book.

### Data collection tools and procedures

Data were collected using standardized, structured and pre-tested questionnaire through face to face interview from April 13 to May 13, 2014. The questionnaire was first prepared in English then translated to local language (Tigrigna) and back-translated into English to maintain the consistency of the questionnaire by local language translator expertise. The questionnaire address socio-demographic characteristics of the patient and client satisfaction questionnaire (CSQ-8) and community interest company questionnaire (CIC) which assess specific providers and service characteristics, which affects satisfaction.

Global satisfaction rate was examined with the client satisfaction questionnaire (CSQ-8), having four responses ranging from poor [1] to very good [4]. Further, specific reasons for satisfaction or dissatisfaction were studied using the modified community interest company (CIC) questionnaire again having four responses ranging from poor [1] to very good [4].

Trained supervisors and data collectors participated in the data collection process from the study area who knows the culture and language of the society. All of the data, collectors were BSc holder in psychiatry and have experience of data collection in mental health survey. Two days of intensive training was given to the data collectors and supervisors on how to conduct the data collection. A brief introduction to data collectors, supervisors and participants had been given before and during the collection process.

### Data analysis

Each completed questionnaire was entered into Epi Info3.5.3 statistical software and then exported into SPSS version 20.0 packages for analysis. Frequencies, proportions and cross-tabulations were used to summarize descriptive statistics of the data. All variables which were p < 0.25 at bivariate analysis were entered into multiple Logistic regression. In multiple Logistic regression to develop the final model, OR was estimated at 95% CI to show the strength of association and p-value < 0.05 was used to declare statistical significance.

### Ethical considerations

Ethical clearance was obtained from the Institutional Ethical Review board of school of public health, University of Gondar. Official permission was secured after communication with Mekelle city administrators through a formal letter. Respondents were informed about the purpose of the study, and written consent was obtained from the study participants so as to maintain confidentiality by omitting their names on the questionnaire and on further dissemination of study result. For those participants who were under the age of consent, assent was taken from their attendants and/or themselves when they were independent minor.

## Results

A total of 415 respondents participated in the study, giving a response rate of 100%. Out of the 415 study participants, 262 (63.1%) were males and 128 (30.8%) were in the age range of 30–39. The mean age of the respondents was 35 (± 12) years. Of the total participants, 278 (67%) were Orthodox, 285 (68.7%) were Tigrian in ethnicity, and 213 (51.3%) were married. Among all study participants, 129 (31.1%) were merchants, 308 (74.2%) were urban residents, 355 (85.5%) had income in the range of ≥ 750 (Table 1).

**Table 1:** Socio-demographic and economic characteristics of patients attending in Mekelle public hospitals, in psychiatry department, Tigray regional state, Ethiopia, August 2014 (n = 415)

Characteristics	Variables	Frequency	Percent
Sex	Male	262	63.1
	Female	153	36.9
Age (years)	< 20	10	2.4
	20–29	116	28
	30–39	128	30.8
	40–49	94	22.7
	≥ 50	67	16.1
Place of residence	Urban	308	74.2
	Rural	107	25.8
Educational status	Informal education	54	13
	Primary education	73	17.6
	Secondary education	93	22.4
	Higher education	195	47
Religion	Orthodox	278	67
	Muslim	73	17.6
	Protestant	24	5.8
	Others	40	9.6
Marital status	Single	93	22.4
	Married	213	51.3
	Others	109	26.2
Ethnicity	Tigre	285	68.7
	Amhara	31	7.5
	Others	99	23.9
Occupation	Government employee	55	13.3
	Private employee	95	22.9
	House wife	40	9.6
	Merchant	129	31.1
	Others	96	23.1
Income	< 750	60	14.5
	≥ 750	355	85.5

Regarding the diagnosis of the respondents, 170 (40.96%) had a psychotic disorder, 127 (30.6%) had mood disorder, 79 (19.04%) had nonpsychosis and 39 (9.4%) had addiction. Only 209 (50.4%) of the respondents had additional general medical condition and 229 (55.2%) had a history of admission at these public hospitals.

Regarding the respondents’ satisfaction, about one-third, (28%) of patients scored in the CSQ-8 “low” satisfaction category (below score 21), whereas 70.8% were satisfied (total score 21–27) and 1.2% very satisfied (total score 27–32). For CSQ-8 items, highest satisfaction rates were quality of service received 315 (75.9%) followed by satisfaction in an overall, general sense, with the service they received 300 (72.3%) and recommending friends if they are in need of similar services 299 (72%). While lowest rates were obtained with a comeback to the hospital to utilize the service (70.8%) and with the amount help they received (70.1%). This study showed that the global satisfaction that is measured by client satisfaction questionnaire (CSQ-8) was 72% to the psychiatric service delivered at the outpatient departments of Mekelle public hospitals, which is the sum of satisfied and very satisfied i.e. the total score between 21–26 and 27–32 respectively.

For CIC items, highest rates were found to their relationship with healthcare staff members (75.7%) and satisfaction with confidentiality and discretion (75.2%) while the lowest rates were observed with the time they waited before their first appointment (50.2%). One hundred fifty-five (37.5%) of the respondents scored in the CIC questionnaire indicated dissatisfied, and the rest 258 (62.5%) were satisfied and very satisfied (Table 2).

**Table 2:** Ranked satisfaction rates for the items of Larson’s CSQ-8 satisfaction questionnaire and the modified CIC satisfaction questionnaire of respondents in Mekelle public hospitals, psychiatry department, Tigray regional state, Ethiopia, August 2014 (n = 415)

CSQ-8 questionnaire	Satisfied % (very good/good)	Not satisfied % (poor/fair)
How do you rate the quality of service you received	75.9	24.1
Did you get the kind of service you wanted	70.6	29.4
To what extent of our health services met your needs	70.8	29.2
If a friend were in need of similar help, would you recommend our service to him or her?	72	28
How satisfied are you with the amount of help you have received?	70.1	29.9
Have the services you received helped you to deal more effectively with your problems?	70.8	29.2
In an overall, general sense, how satisfied are you with the service you have received?	72.3	27.7
If you were to seek help again, would you come back to our hospital?	70.8	29.2
CIC-questionnaire		
Are you satisfied with confidentiality and discretion?	74.7	25.3
Are you satisfied with your relationship with healthcare staff members?	75.7	24.3
Are you satisfied with staff members’ availability?	69.6	30.4
Are you satisfied with the opening hours of this treatment center?	72.3	27.7
Are you satisfied with the length of appointment?	64	36
Are you satisfied with frequency of appointment?	70.6	29.4
Are you satisfied with the information received about your treatment (medication)?	75.2	24.8
Are you satisfied with the time you waited before your first appointment?	50.2	49.6
Are you satisfied with the information received about your health status?	73	27

During the multivariate analysis of psychiatric outpatient service satisfaction in relation to all explanatory variables, educational status, waiting time, type diagnosis were found to be significantly associated.

Those higher educational (diploma and above) were 0.34 times less likely satisfied than the rest educational group (AOR = 0.34, 95% CI 0.24, 0.97). Those complainers of long waiting time were 0.01 times less likely satisfied than those waiting for short time (AOR = 0.01; 95% CI 0.002, 0.07) and those from psychosis outpatient were 2.36 times more likely satisfied than respondents from other outpatients (AOR = 2.36; 95% CI 1.41, 5.72) as shown in (Table 3).

**Table 3:** Association of socio-demographic factors with patient satisfaction among patients getting services in Mekelle public hospitals, Tigray regional state, Ethiopia, August 2014

Characteristics	Level of satisfaction		COR (95% CI)	AOR (95% CI)
	Satisfied (%)	Not satisfied (%)		
Sex				
Male	186 (70.9%)	76 (29.1%)	1	
Female	113 (73.8%)	40 (26.2%)	1.154 (0.737, 1.808)	1.116 (0.70, 1.77)
Age				
18–35	155 (73.8%)	55 (26.2%)	0.892 (0.547, 1.455)	1.064 (0.54, 2.06)
36–45	51 (68%)	24 (32%)	0.754 (0.425, 1.339)	1.159 (0.61, 2.58)
> 45	93 (71.5%)	37 (28.5%)	1	
Marital status				
Single	73 (78.4%)	20 (21.6%)	1	
Married	153 (71.8%)	60 (28.2%)	0.699 (0.392, 1.245)	0.69 (0.88, 1.23)
Widowed/divorced	73 (66.9%)	36 (33.1%)	0.565 (0.296, 1.077)	0.72 (0.29, 1.07)
Diagnosis				
Mood	92 (72.4%)	35 (27.6%)	1.168 (0.534, 2.558)	1.260 (0.56, 2.83)
Psychosis	125 (73.5%)	45 (26.5%)	*2.14 (1.57*, 4.74)*	*2.36 (1.41*, 5.72)*
Non psychosis	55 (69.6%)	24 (30.4%)	1.109 (0.443, 2.341)	1.080 (0.45, 2.55)
Addiction	27 (69.2%)	12 (30.8%)	1	
Educational status				
Non formal education	40 (74%)	14 (26%)	1	
Primary education	57 (79.1%)	15 (20.9%)	1.247 (0.547, 2.841)	1.164 (0.50, 2.66)
Secondary education	68 (73.1%)	25 (26.9%)	0.952 (0.444, 2.04)	0.77 (0.35, 2.1.70)
Higher education	134 (68.7%)	61 (32.3%)	*0.41 (0.862*, 0.87)*	*0.34 (0.24*, 0.97)*
Income				
< 750	48 (80%)	12 (20%)	1.657 (0.846, 3.247)	1.37 (0.68, 2.75)
≥ 750	251 (70.7%)	104 (29.3%)	1	
Waiting time				
< 2 h	209 (74%)	73 (26%)	1	
> 2 h	90 (67.7%)	43 (32.3%)	*0.02 (0.003*, 0.09)*	*0.01 (0.002*, 0.07)**

* Statistically significant at (p < 0.05), ** statically significant at (p < 0.01)

## Discussion

Measurement of patients’ satisfaction in psychiatric hospitals is important because patients’ satisfaction has been correlated with improved hospital administrative measures and quality of care. In addition, measurements of patients’ satisfaction allow organizations to identify areas of service delivery that should be improved.

This paper presents a study that is estimated the level of perceived outpatients’ satisfaction with mental health services at public hospitals in Mekelle Town. According to this study, the satisfaction level of outpatient mental health service utilization at public hospitals in Mekelle town was 72%. This result is lower than study reported from Geneva on patient satisfaction with psychiatric outpatient care, which is 85.1%. This may be due to the accessibility of the facility and diversity in users’ perception [11–13]. On the other hand, a study conducted in Nigeria at Aminu Kano teaching hospital showed that 83% of the respondents were satisfied [14]. The difference could be due to data collection methods and study design. A study conducted in Mozambique showed that the satisfaction level of patients receiving medical care was 55%, which is lower than the finding of this study [9]. Differences may be due to the clinical practice difference across the hospitals.

A study conducted at Jimma hospital showed that the satisfaction level of out-patients users was 57.1%, which is lower than the finding of this study. This could be due to the difference in the nature of the service provided and user characteristics at the hospitals, or it might be partly due to data collection method. The other possible reason for the existing difference could due to well-established administration structure and equipped than the stated hospital on mental health services which has a contribution to the positive satisfaction level of the clients. Since the quality of health service is scaling up currently in Ethiopia, time of data collection may be the other reason.

The satisfaction level of outpatient service consumer in Mekelle public hospitals was related to the educational status of the respondents that shows as the educational level of respondents increases the satisfaction level decreases (AOR = 0.34, 95% CI (0.24, 0.97). This result is in line with findings of a research conducted at Jimma hospital [6]. This shows that patients with relatively higher educational attainment have a greater expectation and they are more critical in analyzing service provided. These could make them less satisfied.

The length of waiting time is found to be significantly associated with the level of respondents’ satisfaction on the mental health services provided at these hospitals (AOR = 0.01, 95% CI 0.002, 0.07). It showed that longer waiting time leads to decrease satisfaction level. This may be related to the number of OPDs, number staffs and the type of service either is more detail or not. Most of the time clients are satisfied with the return home without considering the type of the service. Practically health professionals should consider this issue while they give service.

Another factor that showed significance in this study was the diagnosis of the respondents. Respondents from the psychotic outpatient department were found to be more satisfied compared to respondents from other OPDs (AOR = 2.36; 95% CI 1.41, 5.72). The reason could be due to expectations of the psychotic patients. They could know what the services and they can relate to services given, that makes them more satisfied than the others they do not know the service.

According to this study, the variables: age, sex, marital status, income did not show significant association with the level of satisfaction of the respondents on the service provided at these hospitals. But other studies show that these variables are significantly associated with patients’ satisfaction level. The reason could be due to the difference in the diagnosis of patients receiving health services at these hospitals and possible methodological differences in conducting the researches.

Using the standard tool and primary data are some of the strengths of the study. Social desirability bias might have occurred when completing the questionnaire and difficult to establish a temporal relationship between patient satisfaction and associated factors.

## Conclusion

In this study, the satisfaction level of patients receiving mental health services at Mekelle public hospitals was found to be 72%, which is low and needs improvement. Those with higher education, longer waiting time, diagnosed with Psychosis were found to have a statistically significant association with satisfaction. Improvement in accessibility and availability of drugs, minimizing consultation time (< 45 min) or increasing number of OPD units are important to improve satisfaction. Accessibility and availability of drugs, waiting time, number of OPD units and number professionals should be considered to improve satisfaction.

## Abbreviations

AOR: adjusted odds ratio
BSc: bachelor science
CI: confidence interval
CIC: community interest company
COR: crude odds ratio
CSQ: client satisfaction questionnaire
OPD: out patient department
OR: odds ratio
SD: standard deviation
